# Evaluating ChatGPT’s Accuracy and Readability in Responding to Common Ophthalmology Questions

**DOI:** 10.7759/cureus.87920

**Published:** 2025-07-14

**Authors:** Parsa Riazi Esfahani, Jason Ward, Aidan Yong, Tri Brian Nguyen, Akshay J Reddy, Sina Sobhani, Dalbert Chen, Marib Akanda, Shazia Sheikh

**Affiliations:** 1 Medicine, California University of Science and Medicine, Colton, USA; 2 Medicine, Keck School of Medicine, University of Southern California, Los Angeles, USA; 3 Ophthalmology, Loma Linda University Medical Center, Loma Linda, USA; 4 Ophthalmology, Loma Linda University School of Medicine, Loma Linda, USA

**Keywords:** armd, cataract lens, chat gpt, general ophthalmology, large language models (llm)

## Abstract

Objectives: Eye-related conditions are a prevalent issue that continues to grow worldwide, affecting the sight of at least 2.2 billion individuals globally. Many patients may have questions or concerns that they bring to the internet before their healthcare provider, which can impact their health behavior. With the popularity of large language model (LLM)-based artificial intelligence (AI) chat platforms, like ChatGPT, there needs to be a better understanding of the suitability of their generated content. We aim to evaluate ChatGPT for the accuracy, comprehensiveness, and readability of its responses to ophthalmology-related medical inquiries.

Methodology: Twenty-two ophthalmology patient questions were generated based on commonly searched symptoms on Google Trends and used as inputs on ChatGPT. Flesch Reading Ease (FRE) and Flesch-Kincaid Grade Level (FKGL) formulas were used to evaluate response readability. Two English-speaking, board-certified ophthalmologists evaluated the accuracy, comprehensiveness, and clarity of the responses as proxies for appropriateness. Other validated tools, including QUEST, DISCERN, and an urgency scale, were used for additional quality metrics. Responses were analyzed using descriptive statistics and comparative tests.

Results: All responses scored a 2.0 for QUEST Tone and 1.0 for Complementarity. DISCERN Uncertainty had a mean of 3.86 ± 0.48, with no responses receiving a 5. Urgency to seek care scores averaged 2.45 ± 0.60, with only the narrow-angle glaucoma response prompting an ambulance call. Readability scores resulted in a mean FRE of 45.3 ± 9.98 and FKGL of 10.1 ± 1.74. These quality assessment scores showed no significant differences between categories of conditions. The ophthalmologists’ reviews rated 15/22 (68.18%) of responses as appropriate. The mean scores for accuracy, comprehensiveness, and clarity were 4.41 ± 0.73, 4.89 ± 0.32, and 4.55 ± 0.63, respectively, with comprehensiveness ranking significantly higher than the other aspects (*P *< 0.01). The responses for glaucoma and cataract had the lowest appropriateness ratings.

Conclusions: ChatGPT generally demonstrated appropriate responses to common ophthalmology questions, with high ratings for comprehensiveness, clarity, and support for medical professional follow-up. Performance did vary by conditions, with weaker appropriateness in responses related to glaucoma and cataract.

## Introduction

Large language models (LLMs), like ChatGPT, represent the current advancement in how we interact with and access information [1]. Since its release in November 2022, ChatGPT and other LLMs have reshaped online information retrieval, offering users instant AI-generated responses [2]. These models are increasingly emerging as alternative sources for health-related information, particularly for inquiries about symptoms or medical conditions [3]. However, the growing popularity and use of LLMs raise questions about the accuracy of publicly available online health information, especially regarding vision health [3]. Ophthalmic diseases and conditions affect a significant portion of the global population [4]. The World Health Organization (WHO) reports that at least 2.2 billion individuals worldwide have a near or distance vision impairment, with at least one billion of these cases having been preventable [5]. The prevalence of eye-related diseases continues to grow worldwide, leading individuals to seek available online information regarding the diagnosis, treatment, and symptoms of common conditions, including cataracts, diabetic retinopathy, age-related macular degeneration (AMD), and glaucoma - the leading cause of irreversible blindness, projected to affect 111.8 million people by 2040 [6]. Medical situations in ophthalmology vary in severity, with a multitude of treatments available for conditions ranging from non-emergent issues to life-altering emergencies; therefore, the need for reliable information to ensure patient understanding and informed decision-making is a primary concern. Prior studies have examined the effectiveness of AI and LLM responses to common patient questions in the fields of cardiology [7-8] and urology [9]. While it has been found that these online resources can disseminate expeditious and generally accurate information, current issues persist regarding errors in comprehensive accuracy and newer recommendations generated. To date, there is still a lack of research focused on evaluating the effectiveness of ophthalmology-related searches on LLMs like ChatGPT. Through this study, we aim to examine the accuracy, comprehensiveness, and readability of ChatGPT among the top-searched ophthalmology-related questions by the public. Understanding the current benefits and risks of AI-generated inquiries will have important implications for patient care and decision-making, as well as the need to refine and better control LLM responses for the more effective dissemination of critical medical information.

## Materials and methods

LLM question selection

**Figure 1:**
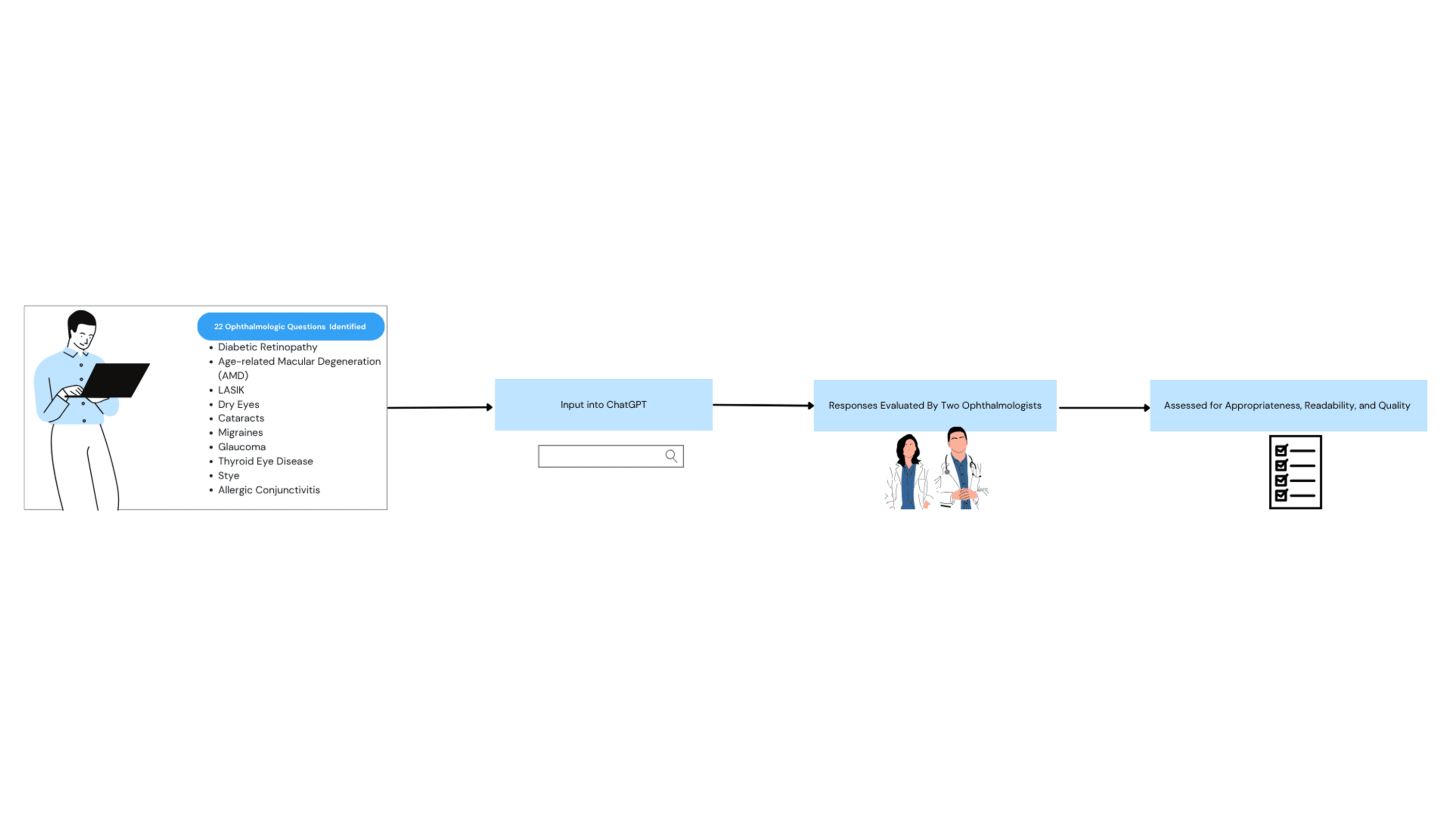
Flowchart of study methodology. Image credit: All authors.

A list of 10 ophthalmologic symptoms and pathologies was inputted in Google Trends (GT) on April 2, 2025 [10]. GT is a Google-based website that displays data on search queries worldwide. There, we identified the top 22 ophthalmologic symptoms most commonly asked on Google. Twenty-two questions were developed and reviewed by members of the research team (PR, AY, JW), one for each symptom (Table 1). The research team certified that each question was clear, clinically relevant, and aligned with the objectives of the study. Team members reviewed to refine the wording, eliminate ambiguity, and confirm that the items accurately captured the intended symptoms. This number provided a feasible yet sufficiently broad dataset for comprehensive analysis. On May 2, 2025, each question was individually entered into a new ChatGPT o1 chat window using the same user account. The first generated responses were compiled into a PDF for analysis by medical professionals (Figure 1).

**Table 1:** Full list of questions inputted into ChatGPT for evaluation. LASIK, Laser-Assisted In Situ Keratomileusis

Question number	ChatGPT input
1	Can diabetic retinopathy cause blindness?
2	Is diabetic retinopathy reversible?
3	What are the Wet macular degeneration symptoms?
4	What is the treatment for dry macular degeneration?
5	What are the LASIK eye surgery side effects?
6	What are the symptoms of dry eyes?
7	What causes cataracts?
8	Can migraines cause blurred vision?
9	How soon can I drive after cataract surgery?
10	How long is the recovery after cataract surgery?
11	How long after cataract surgery can you bend over?
12	What are the restrictions during cataract surgery?
13	Will I need glasses after cataract surgery?
14	Does LASIK hurt?
15	How is glaucoma diagnosed?
16	Can glaucoma be cured?
17	What are the narrow-angle glaucoma symptoms?
18	What is normal eye pressure?
19	Are punctal plugs effective for dry eyes?
20	What is thyroid eye disease?
21	How do I stop a stye early?
22	What is allergic conjunctivitis?

Response assessment

Our methodology was adapted from the study by Davis et al. [9], ensuring a standardized approach to evaluating ChatGPT’s medical responses. Since no validated tool currently exists to assess the quality of LLM-generated medical information, we adapted existing web-based health information evaluation tools to systematically assess ChatGPT’s outputs. The response quality was assessed using multiple validated tools for web-based quality assessment. This includes the Quality Evaluation Scoring Tool (QUEST), DISCERN, a scale from Gilbert et al., Flesch Reading Ease (FRE) formula, and the Flesch-Kincaid Grade Level (FKGL) formula [11-15]. From the QUEST tool, Tone and Complementarity were measured regarding the quality of responses. Tone was scored based on ChatGPT’s responses’ balance and impartiality, while complementarity scores were based on ChatGPT’s responses supporting the provider-patient relationship. DISCERN was utilized to score uncertainty from ChatGPT’s responses. The scale tool from Gilbert et al. [13] was used to score the urgency of responses recommending medical care. Higher FRE scores indicated more readable text, while lower FKRGL scores indicated less readable text. Both FRE and FKRGL scores were measured by inputting generated responses into Microsoft Word (version 2307). An additional question was added to create a binary score on whether ChatGPT urged to seek medical care. Scoring tools were used independently by the two reviewers, who are board-certified ophthalmologists. 

Several validated tools were used to evaluate content characteristics across multiple dimensions. The QUEST tool was applied to assess tone, scored from 0 to 2: a score of 0 reflected fully supported information without acknowledgment of limitations, 1 indicated mostly supported content without discussing limitations, and 2 denoted cautious support that explicitly included discussion of limitations. QUEST was also used to evaluate complementarity, which was scored dichotomously as 0 or 1, with 0 indicating no support for the provider-patient relationship and 1 indicating that the content actively supported this relationship. The DISCERN tool was used to assess uncertainty, rated on a scale from 1 to 5, where 1 represented no uncertainty with clearly defined treatment options, 2 to 4 reflected increasing levels of uncertainty, and 5 indicated definitive uncertainty. To measure urgency to seek medical care, Gilbert et al.’s scale was employed, ranging from 0 to 4: 0 indicated no recommendation to seek care, 1 suggested calling an ambulance, 2 recommended urgent medical attention, 3 suggested non-urgent care, and 4 advised home care. A separate binary variable was also used to capture whether the response explicitly urged the user to seek care, with 0 meaning it did not and 1 meaning it did. Readability was evaluated using two standard metrics: the FRE score, ranging from 1 (non-readable) to 100 (very readable), and the FKGL, where 0-3 corresponds to basic (elementary level), 6-9 to average (middle school), 9-12 to average (high school), and scores above 12 indicate advanced readability (college to postgraduate level).

Ophthalmology assessment of appropriateness of LLM output

Questions inputted into ChatGPT and the corresponding responses were reviewed by two board-certified, native English-speaking ophthalmologists, who rated each response for accuracy, comprehensiveness, and clarity. The areas of uncertainty questions drawn from the DISCERN scale (1-5) were averaged. The ChatGPT responses were independently graded on a 5-point Likert Scale (1: Very Inaccurate, 2: Inaccurate, 3: Somewhat Accurate, 4: Accurate, 5: Very Accurate). ChatGPT Responses graded as 4 or 5 by all ophthalmologists were labeled as acceptable responses. If any ophthalmologist graded a response as a 1, 2, or 3, that response was labeled as unacceptable. A Likert scale of ≥4 in all three subcategories of accuracy, comprehensiveness, and clarity was required for the ChatGPT output to be considered “appropriate.” Each ophthalmologist independently scored each output response and provided reasoning behind their grading. 

Statistical analysis

Descriptive statistics, including mean, standard deviation (SD), and median, were calculated for continuous variables, while frequencies and percentages were reported for categorical variables. Likert scale responses were summarized by reporting the distribution of scores as percentages alongside the median value. Comparative analysis of appropriateness, readability, and quality scores across all 22 questions and relevant subgroups was performed using analysis of variance (ANOVA) for continuous variables, and chi-squared (χ²) or Fisher’s exact tests for categorical comparisons, as appropriate. Statistical significance was determined using two-tailed tests with a *P*-value threshold of <0.05. All analyses were conducted using IBM SPSS Statistics, Version 24.0 (IBM Corp., Armonk, NY), by the Guidelines for Reporting Statistics, Tables, and Figures in Clinical Research in Ophthalmology [16-17].

## Results

The results of 22 ophthalmology-related questions regarding areas of diabetic retinopathy, AMD, LASIK, dry eyes, cataracts, migraines, glaucoma, thyroid eye disease, stye, and allergic conjunctivitis were assessed. Data reported included FRE scores, FKGL scores, tone, complementarity, areas of uncertainty, urgency, recommendations seeking medical care, and the response grading from ophthalmology reviewers. 

Output quality assessment

Based on QUEST Tone and QUEST Complementarity, there were no statistical differences between question responses as Tone (0-2) was rated 2.00 for all responses and Complementarity (0-1) was rated 1.00 for all responses. For DISCERN Uncertainty (1-5), the mean was 3.68 ± 0.48. For Urgency to Seek Medical Care (0-4), the mean was 2.46 ± 0.60. There were no responses graded as a 4 urgency (Home care recommended), and only 1, Narrow Angle Glaucoma Symptoms, was rated as a 1 (Call ambulance). Each of the 22 questions received a QUEST Tone score of 2, meaning each ChatGPT response was deemed to be cautiously balanced but may include statements with identified limitations (Mean = 2, SD = 0, Median = 2). Meanwhile, all 22 responses scored a 1 on the QUEST Complementarity, demonstrating support of the physician-patient relationship. There is no statistically significant difference among major question subgroups for either Tone or Complementarity scores, as all questions received identical scores for these domains. In scoring for areas of uncertainty using the DISCERN scale, seven questions received a 3, while 15 questions received a 4, which averages out to 3.68 ± 0.48. All responses related to LASIK, glaucoma, thyroid eye disease, stye, and allergic complications received a DISCERN score of 4. However, no generated response was awarded a 5 that would correspond with *definite uncertainty*. All questions related to diabetic retinopathy demonstrated the most uncertainty, unanimously receiving a score of 3. There is no statistically significant difference in DISCERN *Areas of Uncertainty* scores between the main subgroups (*P *> 0.05). All individual scores for each condition and related question are listed in Table 2. 

**Table 2:** ChatGPT response assessment via QUEST, DISCERN, Urgency, and Readability metrics. QUEST, Quality Evaluation Scoring Tool

Area	Question	Flesch Reading Ease	Flesch-Kincaid Grade Level	Tone (0-2)	Complementarity (0-1)	Areas of uncertainty (1-5)	Urgency (0-4)	Recommends seeking medical care (Y/N)
Diabetic retinopathy	Diabetic retinopathy blindness	41.3	11.6	2	1	3	3	Y
	Diabetic retinopathy reversibility	21.4	13.3	2	1	3	3	Y
Age-related macular degeneration (AMD)	Wet AMD symptoms	52.9	8.6	2	1	3	2	Y
	Dry AMD treatments	36.3	10.6	2	1	4	3	Y
LASIK	LASIK side effects	45.0	9.4	2	1	4	3	Y
	Pain during LASIK	59.1	7.7	2	1	4	2	Y
Dry eyes	Dry eyes symptoms	60.6	7.2	2	1	3	3	Y
	Punctal plug effectiveness	44.5	10.8	2	1	4	3	Y
Migraines	Migraine blurred vision	51.0	8.1	2	1	4	2	Y
Cataracts	Cataract causes	40.1	10.2	2	1	3	3	N
	Driving after cataract surgery	42.3	11.3	2	1	3	3	Y
	Cataract surgery recovery time	35.7	12.7	2	1	4	2	Y
	Bending after cataract surgery	58.3	8.7	2	1	3	2	Y
	Cataract surgery restrictions	48.9	11.9	2	1	4	2	Y
	Glasses post cataract surgery	46.9	10.6	2	1	4	3	Y
Glaucoma	Glaucoma diagnosis	40.4	10.4	2	1	4	3	Y
	Glaucoma treatment	48.0	9.4	2	1	4	3	Y
	Narrow-angle glaucoma symptoms	42.5	10.5	2	1	4	1	Y
	Normal eye pressure	52.7	9.2	2	1	4	2	Y
Thyroid eye disease	Thyroid eye disease	29.8	12.6	2	1	4	2	Y
Stye	Stye early treatment	60.2	7.3	2	1	4	2	Y
Allergic conjunctivitis	Allergic conjunctivitis	39.1	10.3	2	1	4	2	Y
Mean		45.318	10.109	2	1	3.682	2.455	
Standard deviation		9.979	1.739	0	0	0.477	0.596	
Median		44.75	10.35	2	1	4	2.5	

Urgency and recommendations to seek care

Using the Urgency to seek medical care scale adapted from Gilbert et al., the only question to receive a 1 (recommending calling an ambulance) pertained to symptoms of narrow-angle glaucoma. No response received a score of 0 (no medical care) or 4 (home care recommendations), with a mean score of 2.45 ± 0.60. No statistically significant difference was found in Urgency scores across major question subgroups. The scores were tightly clustered between 2 and 3, with minimal variation among different ophthalmologic conditions (*P* > 0.05). Only in the response to the question about cataract causes did ChatGPT not recommend seeking medical care. For all other 21 question types, response outputs recommend following up with medical professionals. 

Readability assessments

For the readability assessments, the mean FRE score was 45.32 ± 9.98, as shown in Table 2. The output response for diabetic retinopathy reversibility showed the minimum value of 21.4, while the maximum value of 60.6 was observed in the response on dry eye symptoms. All values had a *Z*-score <2, except diabetic retinopathy reversibility. Analysis of FRE scores across major question subgroups demonstrated no statistically significant differences (*P* = 0.275). Although minor variability in readability was observed between conditions, the differences were not substantial enough to reach statistical significance. For FKGL, the mean was 10.11 ± 1.74. The response to Dry Eye Symptoms had the minimum value of 7.2 and the Maximum value was 13.3 with Diabetic Retinopathy Reversibility. All values had a *Z*-score less than 2. The FRE and FKGL scores for each question are listed in Table 2. FKGL scores across major question subgroups did not reveal a statistically significant difference (*P* = 0.100). While minor variability in reading grade levels was observed between conditions, the differences were not substantial enough to achieve significance.

Ophthalmologist expert opinions

Using the Likert scale, the two board-certified ophthalmologists deemed 15 of the 22 (68.18%) responses appropriate using the criteria listed in the methods. Across all evaluated ChatGPT responses, the mean scores were 4.41 ± 0.73 for accuracy, 4.89 ± 0.32 for comprehensiveness, and 4.55 ± 0.63 for clarity. Comprehensiveness scores were found to be rated significantly higher than both accuracy (*P* < 0.001) and clarity (*P* = 0.006), while no significant difference was observed between accuracy and clarity (*P* ≈ 1.0). All ChatGPT responses related to allergic conjunctivitis (1/1), diabetic retinopathy (2/2), dry eye disease (2/2), LASIK (2/2), migraine-related vision symptoms (1/1), and thyroid eye disease (1/1) were judged appropriate. In contrast, only half of the cataract-related responses (3/6) and glaucoma-related responses (2/4) met the appropriateness threshold. Notably, only the responses to questions on driving after cataract surgery, cataract surgery recovery time, and bleeding after cataract surgery received scores of 5 in all three evaluation categories from both ophthalmologists. No response received a score lower than 3 from either ophthalmologist. The individual ratings for each question type are presented in Table 3.

**Table 3:** Ophthalmologists’ Likert scale ratings of accuracy, comprehensiveness, and clarity. AMD, age-related macular degeneration; LASIK, Laser-Assisted In Situ Keratomileusis

Area	Question	Scoring item	MA	DC	Mean
Diabetic retinopathy	Diabetic retinopathy blindness	Accuracy	5	5	5.0
		Comprehensiveness	5	5	5.0
		Clarity	4	4	4.0
	Diabetic retinopathy reversibility	Accuracy	4	4	4.0
		Comprehensiveness	5	5	5.0
		Clarity	4	4	4.0
AMD	Wet AMD symptoms	Accuracy	4	5	4.5
		Comprehensiveness	4	5	4.5
		Clarity	4	4	4.0
	Dry AMD treatments	Accuracy	3	5	4.0
		Comprehensiveness	4	5	4.5
		Clarity	3	5	4.0
LASIK	LASIK side effects	Accuracy	5	5	5.0
		Comprehensiveness	5	5	5.0
		Clarity	5	4	4.5
Dry eyes	Dry eye symptoms	Accuracy	5	5	5.0
		Comprehensiveness	4	5	4.5
		Clarity	5	4	4.5
Migraines	Migraine blurred vision	Accuracy	5	4	4.5
		Comprehensiveness	5	5	5.0
		Clarity	5	5	5.0
Cataracts	Cataract causes	Accuracy	3	4	3.5
		Comprehensiveness	5	4	4.5
		Clarity	5	4	4.5
	Driving after cataract surgery	Accuracy	5	5	5.0
		Comprehensiveness	5	5	5.0
		Clarity	5	5	5.0
	Cataract surgery recovery time	Accuracy	5	5	5.0
		Comprehensiveness	5	5	5.0
		Clarity	5	5	5.0
	Bending after cataract surgery	Accuracy	5	5	5.0
		Comprehensiveness	5	5	5.0
		Clarity	5	5	5.0
	Cataract surgery restrictions	Accuracy	3	5	4.0
		Comprehensiveness	5	5	5.0
		Clarity	5	5	5.0
	Glasses post cataract surgery	Accuracy	3	5	4.0
		Comprehensiveness	4	5	4.5
		Clarity	3	5	4.0
LASIK (pain during LASIK)	Pain during LASIK	Accuracy	4	5	4.5
		Comprehensiveness	5	5	5.0
		Clarity	5	5	5.0
Glaucoma	Glaucoma diagnosis	Accuracy	4	4	4.0
		Comprehensiveness	5	5	5.0
		Clarity	3	5	4.0
	Glaucoma treatment	Accuracy	4	5	4.5
		Comprehensiveness	5	5	5.0
		Clarity	5	5	5.0
	Narrow-angle glaucoma symptoms	Accuracy	5	4	4.5
		Comprehensiveness	5	5	5.0
		Clarity	5	4	4.5
	Normal eye pressure	Accuracy	3	5	4.0
		Comprehensiveness	5	5	5.0
		Clarity	4	5	4.5
Dry eyes (Punctal plug effectiveness)	Punctal plug effectiveness	Accuracy	5	4	4.5
		Comprehensiveness	5	5	5.0
		Clarity	5	5	5.0
Thyroid eye disease	Thyroid eye disease	Accuracy	4	4	4.0
		Comprehensiveness	5	5	5.0
		Clarity	4	4	4.0
Stye	Stye early treatment	Accuracy	3	5	4.0
		Comprehensiveness	5	5	5.0
		Clarity	5	5	5.0
Allergic conjunctivitis	Allergic conjunctivitis	Accuracy	4	5	4.5
		Comprehensiveness	5	5	5.0
		Clarity	5	4	4.5
Mean			4.477	4.754	4.615
Standard deviation			0.734	0.436	0.432
Median			5	5	4.5

## Discussion

In this investigation, the majority of responses (68.18%) were deemed *appropriate* by board-certified ophthalmologists, meeting adequate requirements for accuracy, comprehensiveness, and clarity. These results are similar to previous studies in cardiology and urology that studied the appropriateness of responses from LLMs for patient-generated inquiries [7-9], indicating that general-purpose LLMs can often generate clinically acceptable guidance to patients, although additional studies are needed in other fields of medicine. Certain ophthalmologic conditions and symptom presentations, such as allergic conjunctivitis, diabetic retinopathy, dry eye, LASIK, migraine-related vision questions, and thyroid eye disease, were rated with 100% appropriateness, suggesting that LLMs like ChatGPT can deliver adequate responses to well-defined, frequently discussed topics. However, outputs about cataracts and glaucoma showed only a 50% appropriateness rate. These ophthalmic conditions often involve nuanced surgical decisions and follow-up care that require clinician review and may currently exceed the training depth of the model. Additionally, comprehensiveness was rated significantly higher than both accuracy and clarity, suggesting ChatGPT’s answers tend to be thorough, even when precision or phrasing lags slightly. The tendency for thoroughness to outpace accuracy and clarity is a potentially concerning result of the investigation, which highlights the limit of clinical reliability of LLM-generated content. Patients relying on online health information for urgent or concerning symptoms need confidence that the information being disseminated is not only detailed but also accurate and easily understood.

By tone and complementarity, response outputs remained 100% consistent across all condition subgroups identified. This suggests that ChatGPT maintains a supportive and patient-centered approach in its communication style. Similarly, the DISCERN scale for areas of uncertainty found that there was no statistically significant difference in scoring across question subgroups, highlighting that responses maintained a consistent degree of transparency regarding medical uncertainty. The uniformity of ChatGPT’s supportive tone, patient orientation, and transparent handling of uncertainty across diverse ophthalmic conditions reinforces its potential for broad patient education while minimizing variability in the quality of communicative care. Most outputs in this investigation appropriately recommended that users seek additional medical care when addressing ophthalmologic concerns. All but one response (21/22, 95.5 %) advised seeking evaluation by a healthcare provider, demonstrating strong adherence to promoting physician-patient interactions. Although this high rate is encouraging, getting this value as close to 100% consistency as possible is ideal, given the importance of clinical judgment in patient care and the limitations inherent in AI-generated advice. We further evaluated the degree of urgency conveyed in these recommendations, as ensuring appropriate triage is vital when addressing potentially emergent ophthalmologic conditions. Urgency scores were tightly clustered between moderate values (2-3) across most conditions, suggesting that ChatGPT consistently encourages follow-up without effectively distinguishing between emergent and non-emergent scenarios. Although no overtly inappropriate downplaying of emergent conditions was observed, the uniform urgency across diverse topics raises concern that ChatGPT may not yet reliably prioritize care-seeking behaviors based on condition severity.

Despite the absence of statistically significant differences in FRE and FKGL scores across ophthalmologic topics, the average readability of ChatGPT-generated responses hovered around a 10th-grade level (10.11 ± 1.74). This exceeds the recommended 6th- to 7th-grade reading level for patient education materials, as advised by the American Medical Association (AMA) [18]. Such elevated readability levels may pose comprehension challenges for a substantial segment of the population, considering that only 12% of U.S. adults possess proficient health-literacy skills [19]. The uniformity in readability across diverse topics suggests that while ChatGPT maintains consistent language complexity, it may not adequately tailor its responses to accommodate varying patient literacy levels. Additionally, complex conditions like diabetic retinopathy had higher reading levels, as demonstrated by receiving the lowest score on the FRE scale and the highest FKGL score. Enhancing the readability of AI-generated health information is imperative to ensure accessibility and comprehension across all patient demographics.

As LLMs become integrated into the healthcare workflow, it is essential to establish that medical professionals remain central to patient care and should always be consulted in clinical decision-making. AI and LLMs have great potential to significantly impact healthcare workflow on both the patient and professional side, and therefore need to be evaluated. This study is the first to assess an LLM's ability to generate appropriate and readable ophthalmologic medical information for patient usage. This investigation found that while ChatGPT was able to generate appropriate responses for the majority of ophthalmologic inquiries (68.18%), significant limitations emerged that support the conclusion that patients should ultimately seek care from an ophthalmologist. Specifically, responses to conditions such as cataracts and glaucoma were only rated 50% appropriate, highlighting gaps in the model's ability to handle complex, nuanced conditions that often require individualized surgical or longitudinal management. Moreover, although most responses encouraged users to seek care (95.5%), the urgency ratings clustered narrowly between moderate scores (2-3) and failed to distinguish between emergent and non-emergent situations. This lack of triage specificity is clinically significant, as it suggests ChatGPT may not reliably prioritize care-seeking behavior based on symptom severity. These findings - unlike those from other fields or prior studies - support the conclusion that while ChatGPT may offer general guidance, its limitations in clinical nuance and urgency stratification necessitate follow-up with an ophthalmologist to ensure accurate diagnosis and appropriate treatment. Thus, the recommendation for patients to seek ophthalmologic evaluation stems directly from this study’s observed shortcomings in condition-specific appropriateness and triage guidance, not from extrapolated data in unrelated specialties. While the overall results of this study are relatively positive in regards to LLMs, potential risks are still present. These include subjective visual symptoms (e.g., flashes, floaters, blurriness) being oversimplified by LLMs. Therefore, when given limited user inputs, LLMs could potentially underestimate clinical urgency and disrupt the delivery of timely patient care. Additionally, limited input could overestimate clinical urgency, increasing stress and worry for users. These potential risks highlight the need to develop validated tools for assessing the quality of LLM-specific outputs in ophthalmology and additional medical specialties.

Future directions of this study include fine-tuning LLMs with ophthalmology data to further improve the safety, accuracy, comprehensiveness, and clarity of their responses. Comparative studies evaluating other AI models (e.g., Bard, Bing, Claude, Gemini) would be beneficial to see if quality measures are consistent amongst each other. Lastly, expanding this research to additional medical specialties should be prioritized as LLMs continue to be increasingly utilized in healthcare and society. Primary limitations of this investigation include having a small subgroup sample size, limiting comparison ability, solely medical experts as reviewers with no patient or user perspective, and limited variability in some categories, preventing full statistical analysis. Additionally, with only two ophthalmologists grading responses, discrepancies in scoring can have a disproportionate impact on the results and may not reflect broader consensus within the field of ophthalmology. For example, one ophthalmologist (DC) rated answers ~0.26 points higher on average than MA (*P* = 0.016). Despite statistical significance, the effect size was small, implying a mild leniency bias rather than true disagreement; nonetheless, mixed expertise levels among reviewers could influence perceived appropriateness. Furthermore, LLM model responses were only assessed in English, so our findings do not extend to multilingual patient populations. By progressing with these directions, LLMs can effectively strengthen patient care and healthcare delivery.

Overall, ChatGPT demonstrates a promising baseline competence in ophthalmology, but it also shows condition-specific particularly in cataract and glaucoma care-which underscores the need for targeted refinement and ongoing clinician oversight before routine clinical integration. While LLMs can deliver medically relevant information to patients with rapid turnaround, our investigation highlights lingering concerns, including the omission of critical details for complex conditions and a readability level that may exceed many users’ understanding. While ChatGPT has the potential to enhance access to health information, we acknowledge the concern that its responses may unintentionally contribute to delays in seeking professional medical care, particularly in underserved or remote communities. Patients who receive seemingly reassuring responses might opt to self-monitor or self-treat rather than consult a qualified provider, potentially resulting in worsened clinical outcomes. This underscores the importance of designing AI-generated responses that emphasize the limitations of such tools and explicitly encourage follow-up with licensed professionals. Future iterations of LLMs used in healthcare contexts should incorporate built-in safeguards, such as standardized disclaimers, urgency prompts, and referral language mitigate this risk. As AI continues to be integrated into patient-facing platforms, its deployment must be guided by a principle of complementarity, reinforcing rather than replacing the provider-patient relationship. Future studies should focus on refining LLMs with eye-care-specific data, testing different ways of asking questions, and assessing how real patients understand the answers. Addressing these points will help make LLM output not only helpful but also safe and reliable for patient education and everyday ophthalmic care.

## Conclusions

This investigation highlights the potential role of LLMs like ChatGPT in supporting ophthalmic patient education, with many responses rated as appropriate, comprehensive, and consistently patient-centered. However, several limitations were identified, including reduced appropriateness for complex conditions such as cataracts and glaucoma, a lack of differentiation in triage urgency, and readability levels that may exceed recommended standards for health communication. These findings emphasize that while ChatGPT may be helpful for general guidance on common eye conditions, it should not be relied upon for nuanced clinical decision-making or urgent symptom evaluation. Ongoing development is needed to improve condition-specific accuracy, adjust language complexity for diverse literacy levels, and ensure that AI-generated content aligns more closely with clinical priorities. Future efforts should include fine-tuning with ophthalmology-specific data, involving patient perspectives in evaluation, and expanding research to include multilingual and cross-platform assessments. Until these improvements are achieved, professional ophthalmologic evaluation remains essential to ensure safe and effective patient care.
